# Therapeutic Environments in Drug Treatment: From Stigmatising Spaces to Enabling Places. A Theory-Based Qualitative Analysis

**DOI:** 10.3390/ijerph19095005

**Published:** 2022-04-20

**Authors:** Mads Bank, Kirsten K. Roessler

**Affiliations:** Department of Psychology, University of Southern Denmark, 5230 Odense, Denmark; kroessler@health.sdu.dk

**Keywords:** therapeutic environments, space, stigma, drug treatment, youth, post-structuralism

## Abstract

Investigating therapeutic environments for young drug users is needed to avoid a high dropout rate due to a potential stigmatising effect of the structure of the space. In this article, we draw from three semi-structured interviews with young drug users. The interviews focused broadly on their experiences being on drug treatment and on how they experienced counselling and treatment in different spaces. The findings show that therapeutic spaces that were viewed as clinical and sterile were experienced as stigmatising, which discouraged young drug users from engaging in treatment and therapeutic processes. In contrast, therapeutic places with a homely atmosphere reduced the experience of stigmatisation, facilitated participation in treatment and helped users to relax and feel part of a community. In the analysis, we show how enabling therapeutic places with a homely atmosphere can be produced through materials, activities, and sensory processes.

## 1. Introduction

The purpose of this article is to contribute to our understanding of therapeutic environments through a theoretical analysis of how spaces for individual and group counselling are experienced by young drug users. Investigating therapeutic environments in relation to young drug users is necessary due to the high dropout rate of users in treatment [1,2]. Despite intensive research and development, drug treatment practices face continuing difficulties with getting users to access treatment [3,4,5].

A range of studies indicate that processes of stigmatisation associated with being (labelled as) an illicit drug user are a central factor in explaining problems with adherence and dropout [6,7,8,9]. This shows that stigmatisation processes are complex phenomena [10] working in and across different domains, including individual, group, organisational and structural levels [11].

The literature on drug use suggests that drug users experience multiple types of stigmata [12] that stem from a range of sources including problematic discourses on drug and addiction [13], the general public’s attitudes towards drug users [9], health care professionals’ attitudes [14], and the organisation of treatment and health care [15]. Theories on treatment institutions and stigmatisation in general have shown close connections between stigmatisation, institutional spaces and power [16,17,18]. In the field of drug studies, there is growing interest in contextual and process-oriented ways of understanding ‘treatment’ that include an attention to how different contexts and spaces shape drug use and recovery [19]. How the environmental context and therapeutic spaces play a part in stigmatisation processes in outpatient drug treatment practices has, however, been sparsely investigated [20,21].

The growing interest in the role of space in drug treatment is thematically closely aligned with the significant research in environmental psychology on the relationship between institutional spaces, therapeutic environments and psychophysiological well-being in areas such as hospitals [22], prisons [23] and mental health institutions [24,25].

Studies on in-patient mental health settings [25] have shown how the design of space has an effect on patient–staff interaction [26] and faster improvement [27]. Of particular relevance for the present study is how the configuration space has an effect on the experience of stigmatisation [28] and how symbolic aspects of design can contribute to a feeling of homeliness and promote well-being [29,30]. Acknowledging the broad body of research in the field, we aim to contribute by using a solely post-structuralist approach to analyse the relation between therapeutic spaces, their symbolic meaning, and subjective experiences in drug treatment practices.

A range of authors draw on post-structuralist theory to explore and analyse drug treatment and how spaces [31] or enabling places [32] facilitate recovery [33], how therapeutic and educational spaces modulate subjectivity and participation [20,34,35] and how different places enable persons to abstain from drugs and engage in alternative recreational practices [36]. These studies destabilise the prevailing individualised and psychologised understandings, by replacing ‘the subject’, ‘social context’, ‘drug use’ or ‘drug treatment’ as discrete units of analysis with more relational, contextualised and process-oriented understandings. From a post-structuralist perspective, spaces are produced by heterogeneous materials [37] or interacting social, affective and material forces [38]. Studies have, for instance, analysed how objects, spaces and actants [39] as well as spaces, practices and embodiments [36] produce ‘drug treatment’, ‘stigmatisation’, or ‘recovery’. These post-structuralist analyses allow us to gain a more adequate and complex understanding of how psychological processes and bodily experiences are mediated by spaces and materials in drug treatment practices. The present article contributes to this line of work by exploring an area that has received sparse empirical attention, namely how therapeutic spaces in drug treatment institutions are experienced by and affect young drug users.

### 1.1. Aim

This article aims to contribute to environmental psychology [40] by using post-structuralist theory to analyse how therapeutic spaces—and in particular spaces used for counselling—may contribute to young drug users’ experience of being stigmatised, and how social, material and affective processes can be used to produce more ‘enabling therapeutic places’ that counter stigmatisation and facilitate recovery and development processes.

### 1.2. Theoretical Framework

In this article, we use a post-structuralist, primarily Foucauldian theoretical framework, which implies a relational and processual understanding of spaces, persons and psychological processes [17,18,41]. According to such a view, spaces do not have inherent qualities, but are produced by a composition of heterogenous processes, materials and discourses [38,42,43].

In the present article, our approach aims to analyse what subjects (are able to) experience, do and become in different spaces as an effect of the intertwined processes of (1) the materiality and affective qualities of architecture and space, (2) how spaces are spatialised (through activities and sensory processes) and (3) the subjects’ past and present experiences and ways of being and relating.

These three processes will be analysed by combining a phenomenological understanding of the drug users’ experiences with a theoretical understanding of the significance and affective qualities of spaces [44]

Following the methods section, we shall first show empirically how users experience traditional therapeutic spaces in two Danish drug treatment institutions. Using a Foucauldian understanding of space and power, we will analyse why these therapeutic spaces, contrary to their purpose, enforce stigmatising tendencies and discourage users from treatment. In the second step, we will analyse how counsellors in the same institutions use social, material and affective processes to produce enabling therapeutic places that facilitate development and productive ways of being and relating.

## 2. Materials and Methods

In this article, we draw on empirical material from the research projects ‘User-driven standards in social work’ [34,45] and ‘Aesthetics in drug treatment’ [46]. In both projects, the first author conducted informal and semi-structured interviews [47], participant observation [48], recordings of meetings and collected photos, documents and information from websites to investigate how professionals develop novel approaches for working with young drug users, in ways that counter stigmatisation and move beyond the recovery/harm-reduction dichotomy by focusing on personal development and participation in non-drug-related societal practices. In the first study, the material was organised and coded in Nvivo and the selected parts of the material were transcribed verbatim. In the second study, the first author completed participant observation of how users and professionals experimented with new forms of group treatment, followed by three interviews with users and six interviews with professionals. The interviews were transcribed verbatim. In addition to conducting interviews and making field notes, we collected video material recorded by the participants.

The methodology and analytical approach can be described as a reflexive methodology [49]. In both projects, the empirical material was read by multiple researchers using an open and theory informed coding that allowed themes and analyses to be gradually developed as the researchers reflectively moved between research questions, the empirical material and theoretical resources, drawing especially on post-structuralist theory. Themes of interest and preliminary analyses were continually discussed both among researchers and in ongoing dialogues with the counsellors in the drug treatment institutions in order to validate the relevance of the findings and to select themes to analyse further. In ‘User-driven standards in social work’, some of the major themes were: involving users, user-driven standards, using psychological knowledge and techniques, using technology, governing affect, stigmatisation and the role of space. In the projects on ‘Aesthetics in drug treatment’ [46], some of the main themes were: forms of aesthetic productions, aesthetic processes, motivation, collective/communal, stigma and space. In the present study, we draw on material from 3 semi-structured interviews that are selected because the respondents had particularly interesting and illustrative experiences with what we term ‘disciplinary spaces’ and ‘enabling therapeutic places’.

## 3. Analysis

As mentioned in the introduction, problems with users that do not attend, or prematurely drop out from, drug treatment can partly be attributed to stigmatisation experienced by these users. The empirical material in this article stems from research with treatment institutions that are very sensitive to such problems and continuously try to develop non-stigmatising interventions that accommodate to users’ preferences [45]. Due to this, the empirical material can be regarded as particularly relevant when it comes to analysing the stigmatising and enabling aspects of therapeutic spaces, as the effect of stigmatisation related to other factors can be regarded as diminished.

### 3.1. The Counselling Room and the Office—The Potential Risk of Sterility and Clinical White

The first empirical excerpt stems from the research project on ‘Aesthetics in drug treatment’. The client ‘George’ allowed us to interview him on his impressions and experiences with ‘The Evening Group’. The interview was conducted in a standard room for individual counselling at a Copenhagen drug treatment facility. To the first author, this seemed quite an ordinary space for counselling, decorated in a typical Nordic interior style: white walls with tasteful minimalistic graphic posters, two chairs in a 140 degree angle around a small coffee table. A potted plant, a white/wooden IKEA lamp and the mandatory box of Kleenex on the windowsill. In short, a minimalistic white, neutral, yet tastefully decorated room, similar to many other therapeutic spaces in Copenhagen. During the interview, we came to discuss ‘spaces’ and in particular the contrast between the room for counselling, where the interview was conducted, and the spaces that are used for ‘The Evening Group’.

George: As soon as I entered this room, there’s a whiteboard, and there’s like something clinical about these rooms. (…). It’s insanely white and a whiteboard, and then there’s just some minimalist graphic posters all around. It’s a total museum with modern Nordic decor. I think there should be some sofas and pillows. Almost some dust. Then we wouldn’t have to sit up with our backs straight. But yes, I actually did have a spatial feeling when entering.

I: And how did that affect you? Could you feel it in a way emotionally?

George: It’s almost a hospital atmosphere. Now you’re in a clinical place and it’s becoming formal and almost sacral. Now you’re in a treatment centre!

The next empirical excerpt is from an interview with ‘Molly’ from the project ‘User-driven standards in social work’ (2011–2014). Labelled as ‘a young mum’ (21 years) with illicit drug use at the start of her pregnancy, Molly was a subject of specific concern and was enrolled in multiple interventions. During the interview, we spoke about her experiences with having undergone counselling in different institutions and therapeutic spaces. Below, Molly describes her experience of being in a ‘standard’ counsellor’s office at a traditional treatment centre. The office had both a workstation and place for counselling with two chairs around a coffee table.

Molly: (...) It was very ‘office’-like, so it was difficult to relax because of the computer and the ringing telephone. No matter what you were looking at, you were reminded (…) what this was about. It’s difficult to stop smoking hash and other drugs. Crazy difficult! … And when entering the room, you know that you are at the drug treatment centre and that you have to talk about your emotions (…). When I was here for the first time, I was thinking, ‘I just don’t want to be here!’ The place is so sterile compared to the place I’ve been to before.

If we start with the similarities, both George and Molly experience these spaces as ‘sterile’. For George, the counselling room is ‘insanely white’, looks like a ‘museum’ and is very ‘clinical’, which fits with the cultural standards for using secluded and seemingly neutral spaces for treatment and therapy in the global North. For Molly, the telephone and computer make her associate the room with an ‘office’ that is hard to relate to, and where she does not want to be (for a similar description of white clinical spaces, see [50]). In addition, Molly describes her feeling of being ‘trapped’ in the room, surrounded by office tools, such as a ringing phone. Later in the interview, she also describes how she finds being in the office as ‘psychological with a drab municipal office decor like a typical municipal building’’.

George and Molly both describe how these institutional therapeutic spaces make them reflexively aware of where they are and the ‘treatment’. They describe how they experience bodily discomfort by sitting with their ‘backs up straight’. Molly, in particular, stresses how being in such a space makes it extremely difficult for her to ‘relax and lower her guard’ and engage in therapeutic conversation.

From our perspective, these individual experiences, and the problems they generate for drug treatment practices, are not only explained by individual factors such as personal dispositions, drug use or mental health problems, but are also a consequence of how spaces are intertwined with power. To analyse this, we will introduce a Foucauldian understanding of how spaces play a fundamental role in how disciplinary power governs individuals through processes of normalisation and stigmatisation.

### 3.2. Disciplinary Spaces—Individual Experiences from a Broader Perspective

To Foucault, space and materiality play a far more fundamental role in governing individuals in modern liberal democracies than the well-known vocabulary of discourse and panopticon might lead one to believe [43]. Indeed, to Foucault, ‘Space is fundamental in any form of communal life; space is fundamental in any exercise of power’ [51] (p. 252).

The empirical background for this claim is, in particular, Foucault’s analysis of how the invention and proliferation of disciplinary power relies on a range of institutional spaces such as confession booths, hospitals, classrooms and prisons [18]. At the most abstract level, ‘Discipline organizes an analytical space’ and ‘proceeds from the distribution of individuals in space’ [18] (p. 141), where ‘Each individual has his own place; and each place its individual’ [18] (p. 143). This spatial dispersion makes it possible to assess, judge, compare and evaluate the individual. Through observation and comparison, institutions simultaneously produce a norm for what is considered normal and appropriate and evaluate the individual.

Thus, a fundamental functionality of disciplinary space is that it makes the subject visible as an object of knowledge that can be compared to a norm and be subjected to normalising judgement [18,43]. The psychological mechanism that makes disciplinary power especially effective is that the subject becomes self-reflexive about the normalising judgement and subsequently inverses the ‘disciplinary gaze’ and gradually internalises the task of normalising judgement: Are my behaviour, thoughts and emotions normal and appropriate? How do others see and judge me? How should I behave? These processes are mediated by the culturally available standards, categories and discourses [52].

Foucault describes how disciplinary technologies proliferate and spread across institutions and domains from the 18th century onwards. ‘The disciplines function increasingly as techniques for making useful individuals. Hence their emergence from a marginal position on the confines of society, and detachment from the forms of exclusion or expiation, confinement or retreat.’ [18].

An important point is that disciplinary power is productive and useful. When individuals compare themselves to others through normalising judgement, this facilitates learning and development as they perpetually strive to do their best and develop skills, knowledge and abilities in areas such as education [52,53], sports [54] and leadership [55]. At the same time, discipline makes subjects compliant in relation to existing societal norms and institutional practices. The flip side is that perpetual normalising judgement also produces experiences of personal failure and processes of marginalisation and stigmatisation, as one of the fundamental features of disciplinary techniques is to ‘individualize bodies, diseases, symptoms, lives and deaths’ [18].

### 3.3. The Clinic and the Office—A Sterile Therapeutic Space

Returning to our empirical material, George’s description of the counselling room as ‘insanely white’ and ‘clinical’ with the ‘atmosphere of a hospital’ and giving the impression that ‘you’re at a treatment centre’ is an articulation of how it is experienced from a subject’s perspective when disciplinary power ‘territorialises’ therapeutic spaces [18,56]. In somatic hospitals and other treatment facilities, the white ‘neutral’ clinical space is a disciplinary ‘place of constant, coded, systematic observation’ [57] (p. 56). Here, symptoms and deviances are observed and described as context-independent signs of an underlying individual pathology that can be objectified through a medical diagnosis and treated through professional intervention. In a clinical context, coughing becomes a symptom of an infection rather than polluted air, and drug use becomes a symptom of addiction rather than a coping strategy for handling trauma or difficult life circumstances [58]. When we deal with simple somatic symptoms, these techniques are often efficient and useful.

However, when we are dealing with (young) people in marginal positions and problems associated with stigma, the disciplinary spaces become an obstacle for treatment. Because of the materiality and architectural properties of the therapeutic space, George and Molly find it clinical and sterile and frame what is going on as ‘treatment’. This makes them aware of how these spaces are intertwined with power, as professionals can observe and subject them to ‘normalising judgement’. At the same time, they become increasingly self-reflexive and self-observant. In the interview, Molly articulates this quite clearly:

Molly: You’re more conscious that it, that you are the addict who must sit and talk with the adult professional. (…). Because I was afraid that I’d then get stigmatised, or, . I might say something wrong, or something (…) eh, and you just really feel, you just want to get out of there.

Molly: How can I explain it, well, I, . It’s just a totally different atmosphere.

Although the counsellors work with non-stigmatising approaches, the material configuration of the therapeutic space frames it as disciplinary treatment and the individualising, pathologising and stigmatising tendencies elicit and reinforce the young peoples’ normalising judgement of themselves and makes them feel that there is something wrong with them and their subjectivity. Note that Molly becomes more conscious about her deviant subject position and the stigmatising classification of her being ‘the addict’, which makes her afraid of saying something ‘wrong’. Naturally, this makes her uncomfortable, and she ‘just wants to get out of there’. As mentioned in the introduction, this is not merely a matter of power and space but a consequence of how the materiality and affective qualities of space interact with the subjects’ past and present experiences. As we understand it, Molly’s more pronounced difficulties with the therapeutic space can be attributed to how she, as a young mother, has a more troubled subject position and experiences social stigmatisation to a greater degree than George.

Young people such as George and Molly often have a ‘trajectory’ through educational settings, school counsellors’, psychologists’ and psychiatrists’ offices, where they have been subjected to intensified observation, normalising judgement, sanctioning and other disciplinary techniques. To persons in marginalised positions and with a history of deviance, the disciplinary therapeutic spaces thus become a particular and pertinent problem. Instead of facilitating developmental and therapeutic processes, the ‘disciplinary gaze’ objectifies problems and problematic behaviour in both the present and past and makes the individuals’ deviance even more visible and pervasive. This establishes subject positions to do with deviance, pathology and addiction and contributes to processes of further (self-)stigmatisation and marginalisation.

Having engaged in a Foucauldian ‘negative’ critique of space and power [59], this points to the need for ‘other spaces’ [17]. We do not propose that is it possible to design utopian spaces that are not intertwined with power. Rather, we wish to show how institutional therapeutic spaces can be designed in ways that counter stigmatisation and enable professionals and users to engage more productive social and psychological processes.

In the next section, we will engage in a more affirmative critique [60] and analyse how the materiality and affective qualities of architecture and spaces, together with activities and sensory processes, can be applied to destabilise disciplinary power and produce more enabling therapeutic places.

### 3.4. Enabling Therapeutic Places—The Flat

In the following, we will return to the interview with our interviewee George and his experiences with the therapeutic spaces used for ‘The Evening Group’, which is for young people aged 18–25 who ‘could profit from low-intensive group intervention’. Over the years, there has been ongoing experimentation with how to organise activities in ways that are inviting, helpful and non-stigmatising. In the current structure, a group of two counsellors and five to eight users meet on Thursday evenings for three hours. In parallel to this, users have individual counselling sessions, usually with one of the counsellors running the group. The weekly meetings are held in what used to be a flat on the top floor in an old building, 200 metres away from the main building of the treatment institution in the narrow streets of the old part of Copenhagen’s city centre. The ‘flat’ consists of a large but cramped room, furnished with a conference table, a sofa, a couple of chairs and a small open kitchen. At the far end of the room, there is a ‘wall’ made from glass doors and windows that allow you to peek into two small offices. The flat serves both as a place for working with groups and a meeting room for the counsellors.

George: In the flat, I feel like, . When I arrived there, it was like: all right, it’s nice, calm and cozy. There are these pillars with lots of nails in them, it’s old and it has soul. There’re life in it. It’s not all white and clinical. There are no whiteboards—well, actually, I think there is one, but you hardly even notice it because it’s hidden away.

I: Can you try and say a bit more about how you are affected by that?

George: Yes. You walk into a courtyard, up some narrow stairs to the top floor and when you enter, you smell cooking. It’s informal, relaxed—you don’t have to sit up straight. You can lower your guard. It’s not as formal like ‘now we have to sit here and treat you’. We can just sit here, talk, and have a good time—it’s more like something you want to do.

George describes a quite different experience in what he refers to as ‘the flat’, compared to the counselling room. The atmosphere has changed, given by the material and architectural aspects of the space: The wooden pillars that support the ceiling with visible nails gives the impression that the building is ‘old’, ‘alive’ and has ‘soul’. If we start by paying attention to the material properties, they seem quite straightforward. The building is old and the room has wooden pillars, is cramped with furniture and has a small open kitchen. Altogether, it looks a bit messy, as if it is being used for ‘everyday purposes’. To George, it is ‘nice, calm and cozy’ and resonates with what normally constitutes a ‘homely’ place. The homely place, lacking the sterile atmosphere of the office, was able to contain the individual drug user. In psychoanalyses, the term containing or containment refers to a process where therapists are able to hold the emotions of their patients without reacting to them [61].

In opposition to the office and the counselling room, ‘it’s not all white and clinical’ and does not give the impression of being a hospital or treatment centre; nor does it frame what is going on as treatment. This experience not only affects George’s understanding, but also his affective and corporal ways of being present. He feels he can lower his guard and relax and that he is not being forced to engage in treatment. Rather, as he says, “We can just sit here, talk, and have a good time—it’s more like something you want to do.” Along similar lines, Molly describes the material surroundings at her present treatment centre, which is located in an old villa in a residential area:

Molly: Now you enter a house, but it’s more a feeling of coming home to another person (…) Which it wasn’t over there; it was more like “Well, now you’ve come down to the addiction centre, then you have to sit there and behave properly (…). Here, it’s much more relaxed.

(…) the small things, decoration, pictures, allow me to relax…

(…)

I: Okay, yes.

Molly: It’s more like, . not friendship-like, but more relaxed, it’s less, .Well, it is professional, but it doesn’t feel like that when you come to a place like this, I think.

(…)

I: Okay, that’s very interesting, that it feels differently, even though you know it’s a professional [treatment centre] down here, right?

Molly: Well, that’s the thing, because you’re perfectly aware where you are, but you give it less thought, .

Molly and George recount how spaces that are more ‘personal’ or ‘nice, calm and cozy’ allow them to relax. In a Foucauldian interpretation, this is caused by spaces that are not configured to make the individuals an object of knowledge or have a ‘hard’ disciplinary framing such as treatment. This means that the power of normalising judgement and individualising tendencies becomes less pronounced and less visible. These places, enabling a sense of ‘homely’ and ‘personal’, make the young people less exposed and stigmatised and more relaxed and motivated to attend and participate in treatment.

So far, we have focused on how the material and architectural properties of spaces intersect with (stigmatising) discursive categories, subject positions and young peoples’ past and present experiences. If we want to facilitate participation in treatment, social work and educational practices for people in a marginalised position, this suggests that we should create more enabling therapeutic spaces. Building/modifying the materiality of existing buildings can, however, be quite difficult and expensive.

However, to Molly, the small details—the decor of the room, with candles, pictures, flowers and a wooden buddha statue—already make a big difference and help her to ‘relax in another way’. The small things disrupt the ‘disciplinary gaze’, because they do not constantly remind her that she is at a treatment centre. As we see, this is not because she is unaware that the purpose of the meeting is treatment and a professional conversation intertwined with power. The spatial setup is not made to manipulate her or hide that it is treatment, but rather counter the negative individualising and stigmatising aspects of disciplinary power. This means that being in the therapeutic place feels different, and she can allow herself to relax.

We would now like to turn our attention to how spaces and therapeutic environments are not just a consequence of material properties, but a dynamic and processual accomplishment. Spaces are made, practiced, oriented and made habitable through activities. In the following, we will return to George and ‘The Evening Group’ and elaborate on how the cultural signification and sensory experiences that are produced by preparing and enjoying a meal together re-configure space and change the young persons’ experience of participating in The Evening Group.

### 3.5. Making Therapeutic Places Homely—A Sensory Experience

Over the years, professionals have experimented with different ways of organising sessions in The Evening Group, using diverse activities for framing dialogues and therapeutic conversations. One of the standards that has been established is that to create a sense of togetherness, the group always start by enjoying a communal meal together.

In our understanding, this meal plays an important role in manufacturing and enabling a therapeutic place. George describes the combination of place, activity and sensuous experiences:

George: Yes. You walk into a courtyard, up some narrow stairs to the top floor and when you enter, you smell cooking. It’s informal, relaxed—you don’t have to sit up straight. You can lower your guard. It’s not as formal like ‘now we have to sit here and treat you’. We can just sit here, talk, and have a good time—it’s more like something you want to do.

Additionally, a bit later:

George: Well, it’s this informality—a normalization of it. We’re not in a room designed for just sitting there and being all hospital-like. Like, ‘now we have to treat you’. It becomes mundane. You arrive at this flat, there’s food, and then you just sit and eat, and you don’t talk about hash during dinner but rather about something like, “how was that concert?” It becomes mundane normal, not this situation with one single purpose.

Along similar lines, Sebastian stresses how the meals facilitate a communal aspect and normalisation:

Sebastian: There’s also this communal aspect to it—the shared food. That’s (damn) cozy. I don’t know how to phrase it. It’s difficult. I’m not usually a part of this kind of community’ (…) ‘it’s like an estate agent baking cookies during a house viewing.

Sebastian highlights how (the smell of) food contributes to normalisation and a homely feeling. Spaces and materials (the cobblestoned backyard, the flat, the nails, and the wood) interact with sensory elements (the smell, taste and visual impression of the food) and the cultural signification of the shared food (family, communal, and care) in creating a cosy, pleasant atmosphere. These diverse elements work together and produce a homely place where the young people feel welcome and cared for—an enabling therapeutic place where they can relax, let their guards down and engage in activities and therapeutic processes. When using the example of the estate agent, Sebastian, similarly to Molly, draws our attention to that feeling stigmatised or marginalised or conversely feeling relaxed and being able to let your guard down is not primarily a matter of knowing. The young people know they are participating in treatment, that the flat is not a home and that the group is not their family. However, the particular configuration of space disrupts disciplinary power and reconfigures the young peoples’ experiences and corporeal ways of being. The institutional space has been transformed into a therapeutic place that feels like a home where they can relax, talk and have a good time as a ‘family’—a community or a collective.

## 4. Conclusions

Foucault describes the long European tradition of using specific spaces to facilitate psychological change and transformation and how therapeutic spaces since the 18th century have increasingly become infused with disciplinary power.

In this article, we have critically analysed how therapeutic spaces become an obstacle for treatment and development for young drug users, as these spaces are experienced as disciplinary and reinforce individual experiences of stigma and deviance. Subsequently, we have conducted an affirmative analysis of how spaces, materials, activities and sensory processes can be combined in ways that disrupt disciplinary power and produce more enabling therapeutic places.

We have used post-structuralist theory to understand the relation between space, power and stigmatisation, and our analytical strategy has been to understand individual experiences in therapeutic spaces as an effect of the intertwined processes of (1) the materiality and affective qualities of space, (2) activities and objects and (3) the subjects’ past and present experiences.

Although this article draws on limited material from the field of drug treatment, the findings are relevant to a range of other practices, as problems with stigmatisation, dropout and attendance in drug treatment can be said to be prototypical for practices dealing with people who face social stigma or find themselves in marginalised positions. The findings are a contribution to environmental psychology in general and have relevance for a range of therapeutic, educational, social work and medical practices, where, as Sebastian says, it is important ‘not [to be] in a room designed for just sitting there and being all hospital-like’.
